# From seizures to cognitive dysfunction: A case report of Fahr syndrome in an Afghan patient

**DOI:** 10.1097/MD.0000000000038542

**Published:** 2024-06-21

**Authors:** Shekiba Madadi, Kawsar Alami, Yudai Kaneda, Pazhman Sediqi

**Affiliations:** a Neuropsychiatry Department, Ariana Medical Complex, Kabul, Afghanistan; b Department of cognitive neuroscience, Neuroscience Research Center, Kavosh Nonprofit Educational-Research Institute, Kabul, Afghanistan; c School of Medicine, Hokkaido University, Sapporo, Hokkaido, Japan; d Neuropsychiatry Department, Ariana Medical Complex, Kabul, Afghanistan.

**Keywords:** case report, dementia, Fahr syndrome, hypoparathyroidism, idiopathic basal ganglia calcifications

## Abstract

**Rationale:**

Fahr syndrome is a rare, degenerative neurological condition characterized by bilateral idiopathic calcification of the periventricular region, especially the basal ganglia. This condition is often misdiagnosed as other neurological or psychiatric disorders due to its rarity and overlapping symptoms.

**Patient concerns:**

A 34-year-old man had been experiencing seizures and cognitive dysfunction for few years, which were further compounded by slurred speech and motor difficulties as acute conditions.

**Diagnosis:**

After investigations, severe hypocalcemia, and hypoparathyroidism were detected and his brain computed tomography showed extensive bilateral calcifications in basal ganglia, thalamus, dentate nuclei, and some parts of subcortical white matter, suggestive of Fahr syndrome. Although, the patient was initially misdiagnosed due to a lack of information and the rarity of this disease.

**Intervention:**

The patient was treated with intravenous calcium gluconate, vitamin D3, l-ornithine l-aspartate syrup, and levetiracetam, replacing carbamazepine.

**Outcome:**

His symptoms, including slurred speech, muscle pain, and stiffness improved, serum calcium normalized, and he was discharged with medications for memory deficit and depression.

**Lessons:**

This case underscores the importance of raising awareness among physicians, especially in areas with limited medical resources, about the significance of prompt diagnosis and appropriate symptomatic treatment in enhancing patient prognosis and quality of life.

## 1. Introduction

Fahr syndrome, a rare degenerative neurological condition characterized by bilateral idiopathic calcification of the periventricular region, particularly the basal ganglia, was first described in 1930 by German neurologist Karl Theodor Fahr. The prevalence of this condition is 0.0001% in the general population. It is typically inherited in an autosomal dominant manner,^[1]^ although some cases may be autosomal recessive or sporadic.^[2]^ The most common site of calcification is the globus pallidus in the basal ganglia, but calcification deposits may also be present in other brain regions.^[3]^ The pathophysiology of the calcifications remains unclear, but theories suggest microvesicle disease, disruption of the blood–brain barrier, or neuronal calcium metabolism disorder as possible causes.^[4]^

Basal ganglia calcification occurs in 2 forms: primary basal ganglia calcification, or Fahr disease, which is familial and based on genetic alterations, and secondary Fahr syndrome, which is usually due to secondary causes such as hypoparathyroidism, hyperparathyroidism, or other conditions.^[4–6]^ Fahr syndrome generally appears between the ages of 40 and 60, and its diagnosis is based on clinical manifestations, neuroimaging, and exclusion of primary causes.^[3,7]^ Brain computed tomography (CT) is considered the gold-standard diagnostic technique, with calcified areas appearing as hyperdense lesions on unenhanced brain CT scans.^[8]^ Common symptoms include motor and psychiatric disorders, with psychiatric symptoms present in 40% of patients and seizures being among the rarest manifestations.^[9,10]^

To the best of our knowledge, there is currently no evidence of Fahr syndrome incidence in Afghanistan. However, due to the rarity and overlapping symptoms of Fahr syndrome, this condition frequently encounters diagnostic challenges, often being erroneously identified as alternative psychiatric or neurological disorders. This risk is even higher in Afghanistan, a developing country where access to healthcare remains a significant concern and providing free preventive care and treatment is difficult to achieve. Moreover, the World Health Organization (WHO) has warned of an imminent humanitarian catastrophe unless urgent measures are taken.^[11,12]^ Appropriate diagnosis and treatment for patients with rare conditions like Fahr syndrome can be highly challenging in such circumstances. Thus, providing information and raising awareness among physicians in developing countries, including Afghanistan, is necessary. In this study, we highlight the inaugural manifestation of Fahr syndrome originating from Afghanistan. This case is distinguished by a secondary association with hypoparathyroidism and is characterized by the presence of seizures, early onset cognitive dysfunction, as well as a combination of psychiatric and motor symptoms.

## 2. Case presentation

### 2.1. Patient information and clinical findings

A 34-year-old Asian Afghan man, a resident of Kabul, was brought by his mother in July 2022. His complaints included loss of consciousness, slurred speech, muscle pain and stiffness, weakness in his upper and lower extremities, significant changes in memory and concentration, seizures, and psychiatric symptoms such as depressed mood and negative thoughts. For 8 years, he had been diagnosed as an epileptic seizure patient and his attacks were controlled with carbamazepine (200 mg/day). He was healthy until he began experiencing progressive changes in memory and concentration 2 years ago, 6 years after the initial diagnosis. Over the past 2 months, he has also reported weakness in his upper and lower extremities. Recently, he experienced slurred speech, muscle pain, stiffness, and loss of consciousness leading him to seek medical attention. The patient had no smoking history. Additionally, he had no notable family history. He was admitted to the neurology ward for a thorough examination and treatment.

Upon admission, his vital signs were stable. His blood pressure was 110/80, heart rate 81, oxygen saturation without oxygen mask was 95, and temperature 36.8 °C. He was fully conscious and oriented, with a Glasgow coma score (GCS) of 15/15. He also had slow and slurred speech. His general physical appearance revealed generalized motor weakness, rigidity, and hyporeflexia. A neuropsychological evaluation represents the diagnosis of major depressive disorder with clear clinical evidence of low concentration levels and memory disturbances. Patient did not exhibit any bone abnormalities. Examination of other systems was not significant and remarkable.

### 2.2. Therapeutic interventions

**Figure 1.**
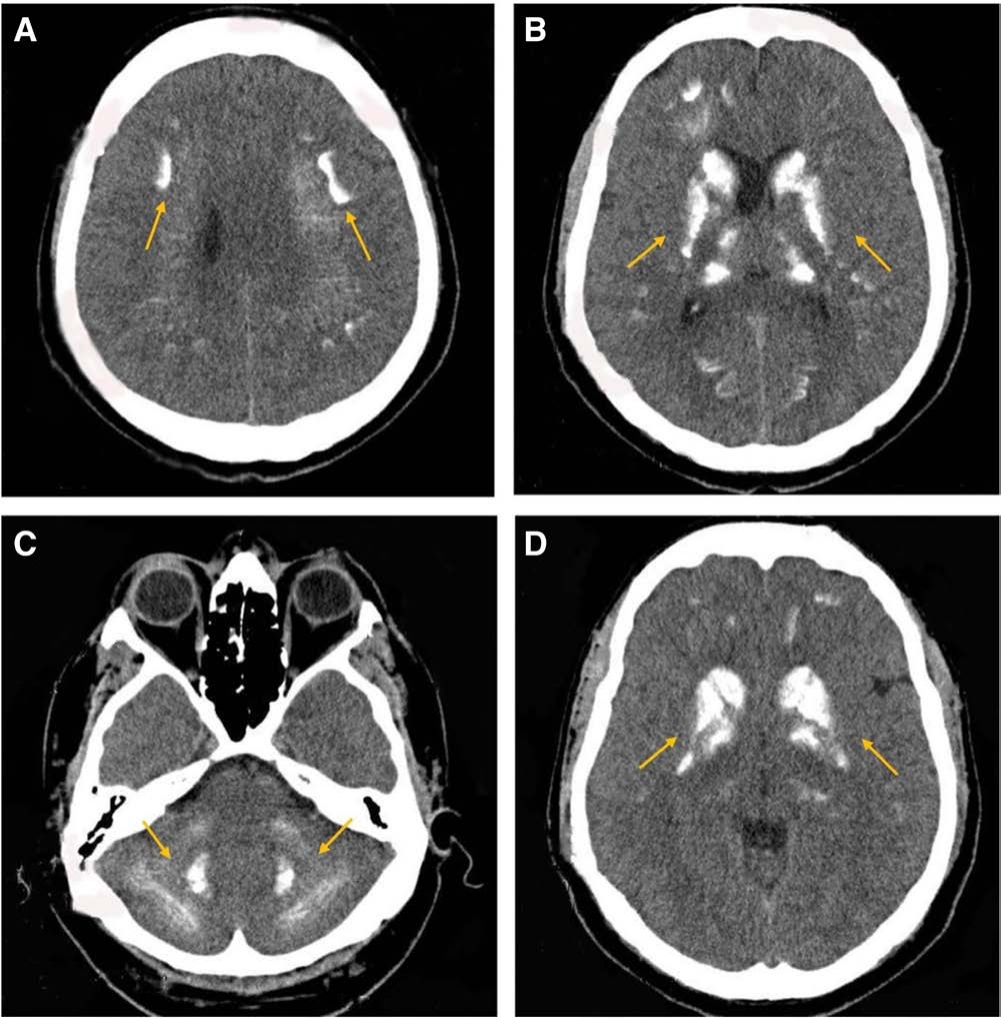
Noncontract CT of the brain showing extensive bilateral symmetric calcification areas in basal ganglia, thalamus, dentate nuclei, and subcortical white matter in axial sections. Arrows show calcification areas.

The blood test results are shown in Table 1. Cell blood count, uric acid, and thyroid stimulating hormone (TSH) were normal. There was an increase in the levels of transaminases (alanine aminotransferase [ALT] and aspartate aminotransferase [AST]: 99 and 59 IU/L, respectively, with normal values being 40 IU/L or below) due to carbamazepine side effects. Electrolytes, including potassium, sodium, and chloride, were normal, but there was severe hypocalcemia (4.8  mg/dL, normal range 8.5–10.5 mg/dL) and hyperphosphatemia (4.7  mg/dL, normal range 2.5–4.5 mg/dL), low 25 (OH) vitamin D (15  ng/mL, normal range 30–70  ng/mL), with a decrease in parathormone (PTH) level (4 pg/mL, normal range 14–65 pg/mL) and normal alkaline phosphatase (ALP) level (62 IU/L, normal range 40–130 IU/L). The patient tested negative for human immunodeficiency virus (HIV), hepatitis B surface antigen (HBs), and hepatitis C virus (HCV). Brain CT showed extensive bilateral symmetric areas of calcification in basal ganglia, thalamus, dentate nuclei, and subcortical white matter (Fig. 1). Unfortunately, due to financial difficulties, the patient was unable to undergo additional tests such as genetic testing and imaging for visualizing parathyroid glands.

**Table 1:** The results of laboratory examinations.

Test name	Result	Unit	Reference range
Calcium	4.8	mg/dL	8.5 to 10.5
Potassium	3.7	mEq/L	3.5 to 5.1
Sodium	144	mEq/L	136 to 145
Chloride	102	mEq/L	98 to 108
Phosphorus	4.7	mg/dL	2.5 to 4.5
PTH	4.0	pg/mL	14 to 65
25 (OH) vitamin D	15.0	ng/mL	30 to 70
Bilirubin total	0.8	mg/dL	Up to 1.0
Bilirubin direct	0.5	mg/dL	<0.5
Bilirubin indirect	0.3	mg/dL	0.3
ALT	99	IU/L	Up to 40
ALP	62	IU/L	Male: 40 to 130 Female: 35 to 105
AST	59	IU/L	Up to 40
TSH	1.69	IU/mL	0.27 to 4.20
Hemoglobin	13.9	g/dL	Male: 13.5 to 16.5 Female: 11.5 to 14.5
TLC	6400	/cm^2^	4000 to 11,000
HCT	42.0	%	Male: 40.5 to 48.0 Female: 34 to 43.0
Platelet count	207,000		150,000 to 450,000
Uric acid	3.0	mg/dL	Male: 3.4 to 7.0 Female: 2.4 to 6.0

ALP = alkaline phosphatase, ALT = alanine aminotransaminase, AST = aspartate aminotransferase, HCT = hematocrit, PTH = parathormone, TLC = total leukocyte count, TSH = thyroid stimulating hormone.

In light of clinical examination, radiological evidence, and the presence of hypoparathyroidism and calcium and phosphorus metabolism disorder, the diagnosis of secondary Fahr syndrome due to hypoparathyroidism was confirmed. The patient was admitted to the critical care unit and treated with intravenous (IV) Calcium gluconate 3 g in 200 mL normal saline every 8 hours as needed and tolerated to the standard ratio, IV vitamin D3 200 IU state (as it has shown to elicit a better response in critically ill patients^[13]^) and l-ornithine l-aspartate syrup. Carbamazepine 200 mg has been replaced with levetiracetam 500 mg, and the patient has been monitored for seizure and liver injury. The liver enzyme levels returned to normal, and no seizures occurred when the patient was hospitalized. After treatment, the serum calcium level was corrected to 5.6 mg/dL, and his acute symptoms, including slurred speech, muscle pain, and stiffness, were resolved. A few days later, the patient was discharged from the neuropsychiatry department with prescribed medicines for his memory deficit (Donepezil) and depressive symptoms (Sertraline).

### 2.3. Follow-up and outcomes

Following the initial assessment, the patient displayed no signs of seizures during the 2-week follow-up period. However, it is important to note that the observed changes in movement and cognitive abilities were progressing at a slower pace. Additionally, there were fluctuations observed in the patient’s blood calcium levels, which have corrected. Unfortunately, during subsequent follow-up appointments, the patient was lost to further evaluation and consultation. It is presumed that financial difficulties might have hindered their ability to attend regular sessions.

### 2.4. Ethical considerations

Ethical committee of Ariana Medical Complex has approved the study and a written informed consent was obtained from the patient to publish this report in accordance with the journal’s patient consent policy.

## 3. Discussion

The following discussion reveals an exceptional occurrence involving an individual in his 30s from Afghanistan. He presented at our hospital with chief complaints of altered consciousness, muscle pain, and psychiatric disturbances. Subsequent evaluations led to a diagnosis of Fahr syndrome, which was determined to be secondary to hypoparathyroidism. Previous studies have reported that the most common cause of Fahr syndrome is hypoparathyroidism, consistent with our case.^[1]^ Also, the brain CT of the patient showed extensive bilateral symmetric areas of calcification in the basal ganglia, dentate nuclei, thalamus, and subcortical white matter. This case confirms the findings of the Ahad et al^[14]^ study that reports bilateral basal ganglia as the most common site of the brain that may be calcified in Fahr syndrome patients. From these aspects, the patient was diagnosed with Fahr syndrome, and treatment was initiated; however, in such cases, where genetic testing is not feasible due to economic reasons, it is crucial to differentiate Fahr syndrome from other causes of brain calcifications, such endocrine and congenital disorders, toxin exposures and infections.^[1,15]^ Endocrine disorders like guanine nucleotide binding protein, alpha stimulating (GNAS) mutation in the form of pseudohypoparathyroidism and mitochondrial encephalopathy with lactic acidosis and stroke-like episodes (MELAS) syndrome, can mimic the signs, symptoms and metabolic changes seen in Fahr syndrome. We ruled out pseudohypoparathyroidism due to resistance to the action of parathyroid hormone with hypocalcemia, hyperphosphatemia, and elevated PTH levels,^[16]^ whereas our patient exhibited a low PTH level. Additionally, MELAS syndrome typically manifests in childhood,^[17]^ which contrasts with our patient’s presentation. While congenital disorders such as carbonic anhydrase deficiency due to tuberous sclerosis complex should be considered for differential diagnosis, the absence of skin hypopigmented macules and patches, or tumors associated with tuberous sclerosis^[18]^ was not observed in our patient. Given the patient’s lack of exposure history to toxins such as lead, mercury, or ionizing radiation, toxin exposure was ruled out as a cause. Infections like toxoplasmosis, neurocysticercosis, or HIV can also lead to brain calcifications,^[1]^ but typically present with different manifestations. Furthermore, the absence of a positive family history may rule out conditions such as interferrinopathies.

The most common and typical clinical features of Fahr syndrome are neurologic symptoms such as seizure, headache, speech impairment, loss of consciousness, and motor symptoms. Fatigability, muscle cramping, dementia, memory and cognition dysfunction, and psychiatric symptoms such as psychosis, depression, and intelligence deterioration have also been seen in some patients.^[1]^ In our case, speech impairment, loss of consciousness, and muscle stiffness manifested as acute symptoms, while the most significant complaints were seizures, depressive and motor symptoms, and particularly cognitive dysfunction. Motor symptoms and psychiatric manifestations such as depression are common and reported in many patients diagnosed with Fahr syndrome.^[10,19]^ However, to the best of our knowledge, seizure is the rarest manifestation of Fahr syndrome and has only been reported a few times.^[9,20–22]^ Another finding in our case was the normal ALP level, despite a low calcium level. This observation may be attributed to the hypoparathyroidism state. Although low calcium levels can stimulate ALP activity, there is evidence indicating that hypoparathyroidism is correlated with normal ALP levels.^[23]^

Furthermore, existing studies have shown that the onset of Fahr syndrome typically occurs within the third to fourth decades of life.^[1]^ We diagnosed the index patient in his third decade of life; however, as the onset of his seizure was 8 years ago, it is possible that the onset of calcifications was in the second decade of his life, which is unusual. The late diagnosis of Fahr syndrome is very common because it is a rare syndrome often misdiagnosed,^[24]^ especially for the young, as we have experienced. The patient we experienced received a timely diagnosis and appropriate treatment at our hospital due to residing in the relatively well-equipped capital city of Kabul and receiving proper support from his family. However, although there have been no previous reports of Fahr syndrome/disease from Afghanistan, it is presumed that a certain number of patients, particularly in remote areas with limited medical resources, may have been overlooked or misdiagnosed.

In particular, this patient’s cognitive dysfunction was quite severe, and according to the family’s account, it significantly impacted the patient’s daily life, making support from those around them indispensable. His memory deficit had a progressive nature that worsened daily for 2 years. Besides this patient, there have been other reports of Fahr syndrome with early-onset dementia and cognitive dysfunction.^[25,26]^ Therefore, when encountering young patients like the one we encountered in this case, who present with symptoms such as seizures and dementia, overlooking the presence of Fahr syndrome/disease due to the lack of guidelines may result in inadequate treatment and poor prognosis. Therefore, raising awareness among physicians to prioritize patients’ health by initially conducting brain CT scans is crucial.^[8]^ Indeed, although predicting the prognosis of Fahr syndrome is challenging, symptomatic therapy typically yields positive results, particularly for secondary forms of the condition. These cases generally have a good prognosis.^[1,14]^ As for the study’s limitations, we were unable to conduct further follow-up on our patient. Therefore, it is crucial to develop a system that can offer continuous and appropriate follow-up care.

In conclusion, this study presents a case involving an individual in his 30s diagnosed with Fahr syndrome in Afghanistan. Hypoparathyroidism is the most well-known cause, and caution is necessary even if there is no family history of Fahr syndrome when symptoms such as seizures and cognitive decline are observed in young patients, as in this case. Since there are no specific guidelines for treating this rare disease, there is a high possibility of misdiagnosis and delayed treatment. Given the potential success of early symptomatic therapy, proactive approaches from physicians, including conducting tests for parathyroid and obtaining brain CT scans, are crucial for the proper diagnosis and treatment of Fahr syndrome.

## Acknowledgments

This study was supported by Ariana Medical Complex (AMC), Kabul, Afghanistan. We thank all officials, especially Dr Abdul Wahed Sidiqi for their valuable support for providing the facilities for thorough investigation and examination of the patient. The authors would also like to thank Dr Matiullah Khurrami for his tremendous help to prepare the manuscript.

## Author contributions

**Conceptualization:** Shekiba Madadi, Kawsar Alami, Pazhman Sediqi.

**Data curation:** Shekiba Madadi, Kawsar Alami, Yudai Kaneda, Pazhman Sediqi.

**Investigation:** Shekiba Madadi, Kawsar Alami, Yudai Kaneda, Pazhman Sediqi.

**Writing – original draft:** Shekiba Madadi, Kawsar Alami.

**Writing – review & editing:** Kawsar Alami, Yudai Kaneda, Pazhman Sediqi.

**Supervision:** Pazhman Sediqi.
